# The ecology of biting: buzzing through the main ecological, environmental and biological drivers of mosquito-borne diseases

**DOI:** 10.1016/j.onehlt.2026.101326

**Published:** 2026-01-12

**Authors:** Elisa Fesce, Josué Martínez-de la Puente, Martina Ferraguti

**Affiliations:** a Department of Veterinary Medicine and Animal Sciences, Università degli Studi di Milano, 26900 Lodi, LO, Italy; b MRC Centre for Global Infectious Disease Analysis, School of Public Health, Imperial College London, London, UK; c Department of Conservation Biology and Global Change, Estación Biológica de Doñana (EBD), CSIC, C/Américo Vespucio, 26, E-41092 Seville, Spain; d CIBER of Epidemiology and Public Health (CIBERESP), Spain

**Keywords:** Insect vectors, Feeding preference, mathematical modelling, Medical entomology, Vector-borne diseases

## Abstract

Mosquito populations are shaped by a variety of environmental drivers, including temperature fluctuations, habitat alterations, and physicochemical factors. These drivers impact mosquito community composition, influencing the spread of vector-borne diseases. Species differ in their sensitivity to environmental changes, with some thriving in anthropogenic landscapes and others exhibit preferences for natural habitats. Abiotic factors such as temperature, water pH, salinity, and dissolved oxygen strongly affect larval survival and development, while interspecific competition among larvae shapes community structure and species abundance, impacting pathogen transmission. Mosquito feeding preferences further influence pathogen transmission by determining host selection; with opportunistic mosquito species that can act as bridge vectors between humans, domestic animals, and wildlife, facilitating the spread of zoonotic pathogens. In this respect, understanding the dynamics of zoonotic pathogens requires a One Health approach that integrates human, animal and environmental health. Mathematical models, in particular, draw on ecological, environmental and biological factors to elucidate mosquito population dynamics and disease transmission, reinforcing the importance of adopting an integrated perspective. We examine the key environmental, ecological, and biological factors shaping mosquito community composition, and highlight the role of mathematical modelling in clarifying how these factors influence mosquito-borne disease transmission. Our findings emphasize that vector surveillance and control programs should target specific vector species in relevant habitats to optimize effectiveness and reduce economic costs.

## Highlights

• Environmental factors like temperature, humidity, and land use shape mosquito communities.
• Mosquito species vary in disease transmission, influenced by biology and ecology.
• Understanding mosquito ecology helps predict outbreaks and improves disease models.
• Integrating ecological and vector data into models aids targeted public health actions.

## 1 Introduction

Vector-borne diseases represent a major global public health challenge, affecting humans, livestock and wildlife. Their incidence and geographic distribution have been expanding, especially in areas such as Europe [1], with vector-borne diseases accounting for more than 17% of all infectious diseases and mosquitoes being among the most widespread vectors [2]. The role of different mosquito species in viral transmission is typically species-specific, with only particular host and vector species demonstrating greater relevance in maintaining and spreading infections [3]. For instance, *Aedes* mosquitoes serve as primary vectors for pathogens causing Chikungunya, dengue, Rift Valley fever, Yellow fever, and Zika, whereas *Anopheles* mosquitoes are responsible for transmitting *Plasmodium* parasites causing human and zoonotic malaria, and O'nyong'nyong virus, among others [4]. Meanwhile, mosquitoes of the *Culex* genus play a crucial role in the transmission of Japanese encephalitis, Lymphatic filariasis, Usutu virus, and West Nile virus, among other [5].

Since 1940, global environmental changes, including habitat alteration, land-use intensification, biotic exchange, the introduction of invasive species, and climate change, have been increasingly linked to the rising incidence and geographic spread of many mosquito-borne diseases [6]. Several of these ecological factors influence the transmission dynamics of these diseases, affecting the absolute abundance of mosquitoes [7], the relative abundance of the most competent vector species, and the composition of host and vector communities [8,9]. Variations in host and vector abundances can modify host-vector contact rates, directly affecting the probability of host infection through mosquito bites [8,10], finally determining transmission patterns of vector-borne diseases [11]. Additionally, climatic characteristics may determine the incidence of vector-borne pathogens with temperature and precipitation playing key roles in the epidemiology of vector-borne diseases [12]. For example, temperature directly affects the transmission dynamics of West Nile virus, where higher temperatures increase the likelihood of virus transmission from mosquitoes to birds [13,14], also affecting its transmission to horses [15]. Also, climatic factors can directly affect incubation periods of pathogens in vectors [16,17], further amplifying their influence on infection dynamics and infection prevalence in host populations [18]. In addition to temperature, other environmental factors, such as rainfall and seasonal variations, can impact mosquito breeding sites and larval survival rates, determining pathogen spread [19]. At a local scale, environmental fluctuations can impact key parameters such as vector feeding behaviour and changes in vector and reservoir host abundance and competence, all of which can modulate the rate of infection in mosquito populations. For example, shifts in host community composition and abundance, driven by intensive land use or wildlife management practices, can significantly impact disease ecology, as hosts affect both vector abundance and pathogen prevalence [20]. Thus, the role of specific wildlife species in maintaining vector and pathogen populations is strongly influenced by the composition of both host and vector communities [21,22]. Therefore, given the complexity of vector-host interactions, disease transmission is shaped by a combination of ecological, environmental and biological factors that are often difficult to disentangle. This complexity makes mosquito-borne infections particularly challenging to study and manage, requiring a deeper understanding of interspecific differences in pathogen amplification across mosquito species requires precise identification of their feeding patterns [23].

In this review, we provide an analysis of the key environmental, ecological, and biological factors influencing mosquito community composition, while emphasizing the importance of mathematical modelling in understanding how these factors affect mosquito-borne disease transmission.

## 2 Environmental drivers shaping mosquito community composition

### 2.1 The role of temperature, habitat alterations, and physicochemical factors in shaping mosquito populations

Mosquitoes, like most insects, exhibit high sensitivity to temperature fluctuations and microclimatic conditions, making them particularly susceptible to the effects of habitat alterations [19,[24], [25], [26]]. Each mosquito species has distinct climatic and habitat preferences [27] and, consequently, responds differently to environmental changes [7]. Environmental factors, therefore, play a pivotal role in shaping mosquito community composition and distribution.

Overall, invertebrate species richness (i.e., the number of different species) and diversity (e.g., measured using Shannon, Simpson, or Evenness indices) tend to decline with increasing levels of anthropogenic land use [28]. However, these effects can be highly taxon-specific [29]. Mosquitoes conform to this general pattern, with vector communities in urban and rural environments typically exhibiting lower species diversity than those in natural habitats [7,30,31]. Nonetheless, exceptions exist, particularly among species with strong invasive potential [29,32,33], which often thrive in urban environments where conditions favour their life cycle development. Examples include species belonging to genera *Anopheles* [34], *Culex* [35] and, particularly, *Aedes* [36], which have successfully adapted to anthropogenic landscapes.

The larval stage of mosquitoes occurs in water bodies, where environmental variables such as nutrient availability, temperature, and pollution influence their survival and development [37,38]. The composition of the larval habitat, including vegetation structure and water chemistry, plays a significant role in shaping the mosquito community's diversity. For instance, certain species exhibit preferences for specific habitat types, leading to variations in community composition [39]. Also, interactions between aquatic larval habitats and surrounding terrestrial ecosystems influence mosquito community composition and abundance, ultimately determining the presence or absence of specific species [40]. Despite the current lack of comprehensive global studies on aggregation behaviour and affinity indices between larvae of different mosquito species, larval co-occurrence and species associations may result from shared breeding site requirements or similar adult feeding habits [41].

Abiotic characteristics of mosquito breeding habitats, particularly physicochemical parameters of aquatic environments, play crucial roles in oviposition site selection, influencing vector community composition and larval density [42,43]. Water turbidity, defined as the cloudiness or haziness of a fluid caused by large numbers of individual particles, has been shown to have significant implications for both water quality and biological processes by modulating water temperature absorbing and scattering light, thereby affecting thermal stratification and impacting larval development [40,44]. High turbidity levels can further reduce the availability of sunlight for photosynthetic organisms, impacting food resources for larvae and lowering search rates for both visual and non-visual aquatic predators, potentially altering community structure [45]. In addition, water temperature is one of the most decisive determinants of larval establishment, growth, and survival across mosquito species [46]. For instance, increasing water temperatures generally accelerate larval development, shorten maturation time, and enhance overall survival. However, optimal survival temperatures do not necessarily coincide with maximal development rates. In *An. gambiae* s.s., a key vector of human malaria in sub-Saharan Africa, survival peaks at temperatures lower than those at which development proceeds fastest [47], highlighting the complex thermal sensitivities that shape mosquito life cycles. These effects on vectors could be also relevant for the transmission of non-human as well as zoonotic malarial parasites such as *Plasmodium knowlesi* [48], *P. cynomolgi* [49], and *P. inui* [50], which are capable of infecting humans under natural or experimental conditions. These cross-species dynamics underscore how environmental drivers of mosquito breeding can influence both zoonotic malaria risk.

Other key environmental parameters, such as pH, salinity, and dissolved oxygen levels in breeding waters, play a crucial role in shaping mosquito community structure and can serve as indicators for predicting the presence of specific mosquito species in an ecosystem [40]. Species-specific responses to these environmental conditions have been documented. Most mosquito species thrive in slightly acidic to neutral pH levels, as extreme pH values can be detrimental to insect survival by influencing the toxicity of various biochemicals in the water [40]. In addition, pH affects the solubility of nutrients and metabolic processes essential for larval development, thereby shaping mosquito community composition. For instance, according to [40], *An. maculipennis* s.l. populations were influenced by pH and turbidity, *Ae. caspius* by alkalinity and temperature, and *Culex mimeticus* and *Cx. modestus* by chloride levels. Additionally, *Cx. theileri* and *Culiseta longiareolata* were affected by electrical conductivity and total dissolved solids, while a strong affinity has been observed between *Cx. mimeticus* and *Cu. longiareolata* [40]. Furthermore, salinity and dissolved oxygen levels have been shown to influence mosquitoes that inhabit brackish waters and can restrict the distribution of certain species, such as *Cx. perexiguus* populations [51]. Therefore, these environmental factors deeply determine mosquito abundance and, potentially, community structure, which can have profound implications for the epidemiology of mosquito-borne diseases [12]. Additional impacts on vector populations could be related to the availability of pollutants in breeding areas, including antibiotics, which could affect the survival rate of mosquito species such as *Cx. pipiens* s.l. [52].

### 2.2 Interspecific competition in mosquito larvae: ecological impacts and methods for quantification

Interactions within mosquito communities alter mosquito-pathogen relationships and shape the spread of diseases [53]. These dynamics, such as competition for limited resources, occurs both within species (intraspecific competition) and between species (interspecific competition), and mosquitoes are no exception to these ecological dynamics. Interspecific competition primarily occurs at the larval stage, significantly influencing the composition and structure of mosquito larval communities [54,55].

Different studies have been conducted using different vector species in this respect, demonstrating that *Ae. albopictus* larvae are highly competitive with other mosquito species cohabiting the same breeding sites. Interestingly, the presence of *Ae. albopictus* has been shown to negatively impact the growth and survival of other species including *Ae. aegypti* [56], *Ae. cretinus* [57], *Ae. japonicus* [58], *Ae. koreicus* [59] and *Cx. pipiens* s.l. [60,61]. However, the opposite pattern is not always observed, as *Ae. albopictus* larvae appear largely unaffected by the presence of *Cx. pipiens* s.l. larvae. This asymmetric interspecific competition is often attributed to differences in the efficiency of resource conversion into biomass, reproductive output, or competitive dominance under limiting conditions [62]. Moreover, the intensity of competitive interactions depends on environmental factors such as resource availability and temperature. For instance, the highest competition-induced mortality in *Cx. pipiens* s.l. larvae has been recorded at temperatures exceeding 25 °C [62]. As a consequence, interspecific interactions at the larval stage have profound consequences for adult mosquito survival and population dynamics [63,64]. Given the potential role of sympatric species in the transmission of vector-borne diseases worldwide, further research is essential to deepen our understanding of these complex dynamics.

In the context of interspecific competition among mosquito larvae, the degree of affinity between pairs of *Culicidae* mosquitoes within the same habitats can be quantitatively assessed using Fager and McGowan's affinity test [65] and affinity index (see the Formula below). This index ranges from −1 (complete dissociation) to +1 (complete association), with values of 0.5 or greater considered indicative of strong affinity [66]. This threshold was chosen to categorize species as co-occurring when they are found together in more than 50% of their recorded occurrences, suggesting that they likely share similar environmental requirements. As a result, the presence of one co-occurring species increases the likelihood of the second species' presence. The significance of the affinity index is typically assessed using a *t*-test, with a standard significance level set at 5% [67,68]. High affinity and association among mosquito species indicate co-occurrence, suggesting that these species share similar habitat requirements that influence their larval development and biology. These shared needs may lead to competition for resources such as food, exposure to predators, and increased vulnerability to pesticides.

**Table:** 

	Formula
Affinity index	$I=\left[\frac{J}{{\left(\mathrm{nA}+\mathrm{nB}\right)}^{1/2}}\right]-\left[\frac{1}{2{\left(\mathrm{nB}\right)}^{1/2}}\right]$
Parameters⁎	*J* is the number of joint occurrences, *nA* is total number of occurrences of species *A*, *nB* is total number of occurrences of species *B.*
“*t*” test	$t=\left[\frac{\left(\mathrm{nA}+\mathrm{nB}\right)\;\left(2J-1\right)\;}{\left(2\mathrm{nAnB}\right)}-1\right]\left[\sqrt{\left(\mathrm{nA}+\mathrm{nB}-1\;\right)}\right]$

⁎ *Species are assigned to the letters so that nA < nB.*

## 3 Mosquito feeding preferences and their impact on pathogen transmission

Blood feeding patterns of mosquitoes determine the contact rates between infected and susceptible hosts of pathogens [69]. The frequency of mosquito bites is especially critical in shaping the magnitude of pathogen epidemic peaks in mosquito populations, such as in the case of West Nile virus (WNV) [70]. Additionally, biting frequency can influence pathogen transmission rates and, consequently, the incidence of various diseases [69], with its impact being particularly critical in shaping the magnitude of WNV peaks in mosquitoes [70]. Increased biting rates can enhance pathogen encounter rates with suitable hosts, thereby enhancing pathogen transmission probability and frequency, and boosting pathogen prevalence within the community. In this context, for the case of zoonotic pathogens, vector feeding behaviour directly connects wildlife, domestic animals and humans, positioning mosquito–host interactions at the core of a One Health framework.

Different approaches have been developed to identify the blood-feeding patterns of mosquitoes and other insect vectors. Recent advances in molecular techniques, such as species-specific and universal primers, have significantly improved our ability to identify mosquito feeding preferences by amplifying host DNA from recent blood meals [71]. These methods enable researchers to determine mosquito host species at a fine scale, enhancing our understanding of host-vector interactions. Additionally, further molecular analyses have allowed for individual-level identification of vertebrate hosts of mosquitoes through the amplification of highly polymorphic DNA regions, such as microsatellites [72] or differences in the blood feeding patterns between hosts of different sexes [73]. By revealing heterogeneities in disease transmission and identifying the most efficient vectors and reservoir hosts responsible for pathogen spread, these studies provide crucial insights into the epidemiology of vector-borne diseases. They may also help identify key species that function as superspreaders, revealing how pathogens move across wildlife reservoirs, livestock, and human populations [10,74].

Current evidence supports that mosquito species exhibit innate selective feeding preferences, with variation in feeding tendencies at different levels. Some species feed primarily on mammals (mammophilic species), such as *An. atroparvus, Cx. theileri,* and *Ae.* (*Ochlerotatus*) *caspius*, while others show a preference for birds (ornithophilic species), including *Cx. perexiguus, Cx. pipiens* biotype *pipiens,* and *Cx. modestus* [75]. Some species may even feed on amphibians and reptiles [[76], [77], [78]]. In addition to these broad host taxa, mosquitoes feed on specific host species at higher rates than expected based on host abundance [23,79]. For instance, *Cx. pipiens* biotype *pipiens* demonstrates a preference for feeding on blackbirds (*Turdus merula*) compared to European starlings (*Sturnus vulgaris*) [72], while avoiding hirundine species [74]. However, mosquito feeding preferences are still influenced by host availability according to the host community composition, which varies according to seasonality and habitat potentially explaining the incidence of WNV in human populations [10,80]. Indeed, to ornithophilic species, *Cx. pipiens* biotype *pipiens* and *Cx. tarsalis*, may shift their diet to include more mammals when the availability of certain bird species decreases towards the end of summer, thereby affecting the transmission of pathogens from birds to humans [10,81]. These shifts of mosquito feeding patterns according to the availability of vertebrate hosts illustrate how ecological changes in wildlife communities can rapidly alter human exposure risk, reinforcing the interconnectedness emphasized by One Health principles.

### 3.1 Major recognized mosquito vectors in Europe and their feeding habits

**Fig. 1:**
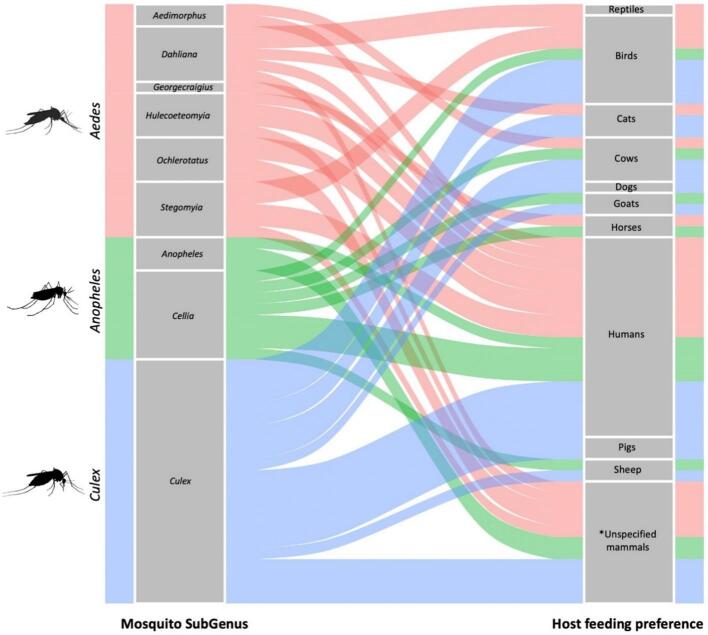
Alluvial plot showing mosquito and host feeding preference networks for mosquito species of medical importance in Europe following Wilkerson et al. [5]. The flow lines represent the different relationships between mosquito species and the vertebrate source they feed on. In this plot, the left column represents vector species, while the right column represents host identity, which are embedded in the flow of the plot. Different colours represent the mosquito genus where *Aedes* are shown in red, *Anopheles* in green, and *Culex* in blue. The width of the bands connecting the categories reflects the relative number of observations moving in the network. *Note*: The host feeding source species can be repeated in the right column, reflecting the fact that different vector species can feed on the same vertebrate. (For interpretation of the references to colour in this figure legend, the reader is referred to the web version of this article.)

Mosquito diet composition has been identified as one of the most critical factors influencing transmission risk [70,82]. Mosquitoes of the genus *Culex*, particularly *Cx. perexiguus*, serve as the primary vectors in amplifying and sustaining WNV transmission in southern Spain, by preferentially feeding on highly competent avian hosts [83]. Using information on the feeding preferences of the most significant mosquito vector species in Europe from a public health perspective, as well as their biting frequencies and relevant ecological traits obtained Mosquitoes of the World book [5], it was possible to identify at least 19 vector mosquito species present in Europe, spanning nine subgenera within the *Aedes*, *Anopheles*, and *Culex* genera [84]. These species are globally recognized as significant vectors of 135 medically relevant pathogens, including bacteria, fungi, nematodes, protozoa, and viruses (see Table 2 in [5,84]). An analysis of their feeding preferences reveals considerable heterogeneity in host selection among mosquito species, as demonstrated at the species level (Fig. 1). On average, mosquito species feed on three different vertebrate hosts, with a range of 1 to 9 species (1). Notably, humans were identified as hosts for all mosquito species except *An. maculipennis* s.l. However, species of this complex are known to be human-blood bitters [85], suggesting their role in the transmission of human pathogens in Europe.

Interestingly, all species showed either predominantly mammophilic or opportunistic feeding behaviours, with avian hosts always present in conjunction with mammalian species, further underscoring the potential for interspecific host-connections, considering species of different taxa. Understanding these multitrophic mosquito–host networks is therefore essential for predicting cross-species pathogen flow. Among the species analysed, *Cx. theileri* exhibited the broadest host range, feeding on nine different vertebrate species, followed by *An. sergentii* (*n* = 6), *Ae. geniculatus* (*n* = 5), and *Cx. quinquefasciatus* (*n* = 4). The remaining mosquito species fed on fewer than three vertebrate hosts (Table 1). On the contrary, *Ae. atropalpus, Ae. koreicus, An. multicolor,* and *An. superpictus* were the most specialized species, feeding exclusively on humans (Table 1). This host specificity is particularly relevant given their potential role in transmitting critical pathogens, such as Japanese encephalitis virus, WNV, St. Louis encephalitis virus, and Rift Valley fever virus [4,5].

**Table 1:** Summary of mosquito host-feeding preferences across vertebrate taxa, based on data from *Mosquitoes of the World* [5]. Host categories include reptiles, birds, and a range of mammalian species. For mammals, specific hosts are detailed when available.

Mosquito species	Reptiles	Birds	Mammals									
			Cats	Cows	Dogs	Goats	Horses	Humans	Pigs	Sheeps	Unspecified mammals	Tot
*Aedes (Georgecraigius) atropalpus*								1				1
*Aedes (Hulecoeteomyia) koreicus*								1				1
*Anopheles (Anopheles) maculipennis* s.l.											1	1
*Anopheles (Cellia) multicolor*								1				1
*Anopheles (Cellia) superpictus*								1				1
*Aedes (Aedimorphus) vexans* s.l.				1				1				2
*Aedes (Ochlerotatus) caspius*								1			1	2
*Aedes (Ochlerotatus) dorsalis*								1			1	2
*Aedes (Stegomyia) aegypti*		1						1				2
*Anopheles (Anopheles) claviger*								1			1	2
*Aedes (Hulecoeteomyia) japonicus* s.l.							1	1			1	3
*Aedes (Stegomyia) albopictus*		1						1			1	3
*Culex (Culex) perexiguus*		1						1			1	3
*Culex (Culex) pipiens* s.l.		1						1			1	3
*Culex (Culex) tritaeniorhynchus*				1				1	1			3
*Culex (Culex) quinquefasciatus*		1						1			2	4
*Aedes (Dahliana) geniculatus*	1	1	1					1			1	5
*Anopheles (Cellia) sergentii*		1		1		1	1	1		1		6
*Culex (Culex) theileri*		1	2	1	1	1		1	1	1		9

### 3.2 Implications of mosquito feeding strategies for pathogen spread: specialists vs. generalist

The specificity of mosquito feeding behaviour can have significant implications for pathogen spread. If a vector is non-selective and feeds on a broad range of hosts, the pathogen may benefit from being a generalist, maintaining compatibility with various vertebrate species, even if some are suboptimal hosts. This broader feeding variety increases the pathogen's encounter rate with suitable hosts, thereby boosting its overall prevalence [86,87]. Conversely, if a vector feeds on a specific vertebrate species, the pathogen may benefit from being a host specialist, improving transmission within a narrower host range [81,88]. Opportunistic mosquito species that frequently feed on both mammals and birds play a key role as bridges between species, facilitating the transmission of zoonotic pathogens [74]. That is the case of species of the genus *Culex*, particularly *Cx. pipiens* s.l. and *Cx. perexiguus*, that exhibit opportunistic feeding behaviours. While typically considered ornithophilic, these species have been shown to feed on humans and horses [23,82].

Recent epidemiological models have demonstrated the differential contributions of several mosquito species in the transmission of vector borne pathogens, including those affecting wildlife. For the case of zoonotic pathogens such as WNV, Ferraguti et al. [89] estimated the basic reproductive number (*R*_*0*_) in areas with different mosquito species, showing the varied contributions of each vector species to the WNV transmission cycle. *Culex perexiguus* was identified as the most significant species in amplifying WNV in southern Spain. Contrary to the case of WNV, in the case of avian malaria parasites, which infect birds but not mammals, *Cx. pipiens* s.l. were identified as the vector with higher *R*_*0*_ values, either alone or in combination with *Cx. modestus* or *Cx. perexiguus*. These results highlight the importance of vector species identity in mathematical modelling of vector-borne diseases [84], underscoring the need to consider the overall composition of the insect community, their feeding patterns, and vector competence when studying disease transmission [89,90].

## 4 Mathematical modelling of ecological and biological factors in mosquito population

Following the seminal work of Ross [91,92], numerous models have been developed to investigate the role of mosquitoes in disease transmission and emergence. The integration of statistical techniques for analysing emergence patterns with ecological relationships between biotic and abiotic factors, alongside mathematical modelling, represents a significant advancement in disentangling the mechanisms underlying infection transmission and spread.

Mathematical models, in particular, explicitly simulate infection dynamics over time, incorporating biological processes such as interspecific competition, temporal niche effects, and variations in feeding preferences. Given the critical role of vector population dynamics in infectious disease transmission, these models have primarily been used to describe the population dynamics of key mosquito species, such as *Ae. albopictus* [[93], [94], [95]] and *Cx. pipiens* s.l. [61,96]. Additionally, mathematical models have investigated larval competition, focusing on temporal niche effects and asymmetric interspecific competition between *Ae. albopictus* and *Cx. pipiens* complex in Italy [97]. These models demonstrate how environmental variables such as temperature and photoperiod influence the timing of peak mosquito abundance. They also predict that *Cx. pipiens* larval mortality increases in the presence of *Ae. albopictus* within the same breeding site in temperate climates, leading to decline of *Cx. pipiens* s.l. adult populations. Moreover, drier weather conditions may promote greater overlap of breeding sites, intensifying interspecies competition. These findings underscore the importance of modelling in understanding the interplay between climate, competition, and vector diversity, which is crucial for assessing epidemiological risks and anticipating mosquito disease risks across human, animal, and environmental components [98,99].

Theoretical studies highlight the significance of mosquito feeding behaviour in pathogen transmission [70] and explore how different transmission assumptions influence predictions of WNV spread [100]. For example, Fesce et al. [70] showed that an extended viraemic period in birds increases their infectiousness, thereby raising the likelihood that mosquitoes feed on infectious hosts (and vice versa), ultimately amplifying transmission. A comparable effect arises from higher mosquito biting rates on competent bird species, which increase the probability of encounters between susceptible mosquitoes and infectious birds (and vice versa). In addition, the study by Wonham et al. [100] demonstrated how model structure shapes expectations about the effectiveness of control measures. Under frequency-dependent transmission, reducing vector density lowers outbreak risk, whereas reducing reservoir host density may unintentionally increase it. In contrast, under mass-action transmission, where low densities of vectors and hosts limit biting rates, reductions in either population are predicted to decrease the probability of disease emergence. Several models assess the efficacy of intervention strategies against major mosquito-borne diseases, emphasizing the importance of proper vector management to mitigate WNV spread [[101], [102], [103]]. Additional models also evaluate the impact of vaccines and diagnostic testing [[104], [105], [106]], emphasizing the need for integrated approaches that consider both vector ecology and the effectiveness of interventions to optimize disease control.

Additionally, models have been used to examine the potential impacts of climate change on vector-borne diseases, projecting how environmental shifts may alter transmission patterns. For example, Bomblies and Eltahir [107] showed that changes in precipitation patterns, especially shifts in the frequency and timing of rainfall events rather than total rainfall, can nonlinearly alter mosquito abundance and, given that vectorial capacity does not scale proportionally with disease prevalence, lead to complex and disproportionate impacts on mosquito-borne disease transmission. In malaria control and elimination efforts, models facilitate the tailoring of interventions to local contexts, demonstrating the importance of targeting high-transmission hotspots to maximize resource efficiency [108]. In the case of dengue, mathematical models have been crucial for understanding how fine-scale human movements, in conjunction with vector behaviour, influence transmission dynamics. They showed, for example, that key transmission sites are not necessarily those with the highest vector abundance, as is often assumed, but also those where people spend most of their time [109]. Models exploring antibody-dependent enhancement (ADE) in multiserotype dengue infections reveal that ADE can extend inter-epidemic periods and allow low-transmission strains to persist by relying on higher-transmission strains [110]. Here, they also provide insights into epidemic phase relationships and the stability of transmission dynamics, showing that if cross-reaction is present, as in the case of dengue, epidemiological dynamics are likely to be influenced by positive feedback between the intensity of cross-reaction and epidemic outcomes [111]. Furthermore, integrating ecological and immunological mechanisms within theoretical models has advanced our understanding of multiannual cycles and serotype persistence, suggesting that seasonality is essential to account for intra-annual variation in monthly dengue incidence, though it plays a smaller role in shaping interannual dynamics. Additionally, a minimum duration of cross-immunity is required to reproduce the empirically observed three-year epidemic cycles [112].

Beyond biological and ecological factors, a substantial body of modelling research integrates vector population dynamics with pathogen transmission. Here, mathematical and computational models are critical for understanding disease dynamics and designing effective public health strategies due to collectively, these models provide invaluable insights into disease transmission and control, serving as essential tools for managing vector-borne diseases. They inform strategies for climate change adaptation, localized disease control, and the development of effective interventions, ranging from vector management to vaccination and diagnostic testing. Importantly, these modelling frameworks increasingly adopt a One Health perspective by explicitly linking mosquito ecology, wildlife reservoir dynamics, livestock exposure, and human infection risk, providing integrated tools for cross-sectoral decision-making.

## 5 Concluding remarks

Vector life history traits and their ecological, environmental and biological requirements are crucial for determining the incidence and distribution of vector-borne diseases. Variation between vectors in how they spread disease, particularly through their impacts on pathogen host range and effects on transmission rates, is a key factor to consider in control and surveillance programs. Temperature, among other climatic conditions, and habitat alterations influence mosquito life history traits, survival, and distribution, which in turn affect disease dynamics. Interspecific competition among larvae, particularly in response to environmental factors, shapes mosquito populations and their capacity to transmit pathogens. Given the high environmental variability affecting mosquito establishment and spread worldwide, it is essential to determine the ecological and biological requirements of larval habitats, as well as species-specific associations of medically important mosquitoes. Also, feeding behaviour, particularly mosquito preference for specific hosts, further complicates the transmission of zoonotic pathogens, with generalist species potentially acting as bridge vectors between humans, domestic animals, and wildlife. Identifying the biotic and abiotic factors affecting mosquito breeding and host selection can help map oviposition sites, vector distribution, and identify vertebrate hosts, facilitating targeted surveillance and control programs. Mathematical modelling is essential for simulating these dynamics, assessing the impact of climate change and vector management interventions, and optimizing public health strategies. Future research should expand models to incorporate finer-scale ecological variables and further explore how climate change influences mosquito behaviour, pathogen transmission, and human–animal–environment interactions.

## Data accessibility

No unpublished data are presented.

## CRediT authorship contribution statement

**Elisa Fesce:** Writing – review & editing, Visualization, Data curation. **Josué Martínez-de la Puente:** Writing – review & editing, Supervision, Conceptualization. **Martina Ferraguti:** Writing – review & editing, Writing – original draft, Visualization, Software, Project administration, Methodology, Investigation, Funding acquisition, Formal analysis, Data curation, Conceptualization.

## Ethics

This work involved no human subjects.

## Funding

This work was partially supported by projects PID2022-142803OA-I00 from the 10.13039/501100004837Spanish Ministry of Science and Innovation and a 2023 Leonardo Grant for Researchers and Cultural Creators (LEO23-2-10078, 10.13039/100007406BBVA Foundation) to M.F. The 10.13039/100007406BBVA Foundation accepts no responsibility for the opinions, statements and contents included in the project and/or the results thereof, which are entirely the responsibility of the authors. MF is currently funded by a Ramón y Cajal postdoctoral contract (RYC2021-031613-I) from the 10.13039/501100004837Spanish Ministry of Science and Innovation (MICINN). EF is supported by MUSA – Multilayered Urban Sustainability Action – project, funded by the 10.13039/501100000780European Union – NextGenerationEU, under the National Recovery and Resilience Plan (NRRP) Mission 4 Component 2 Investment Line 1.5: Strengthening of research structures and creation of R&D “innovation ecosystems”, set up of “territorial leaders in R&D”.

## Declaration of competing interest

We have no competing interests.

## Data Availability

Data will be made available on request.
